# Annual and Seasonal Trends in Urine Drug Screening Positivity: A Four-Year Retrospective Study at a North Indian Tertiary Care Hospital

**DOI:** 10.7759/cureus.114049

**Published:** 2026-08-06

**Authors:** Shantanu Dey, Manish Gupta, Mansi Tomar, Pankaj Arora, Simran Sein, Gurnoor Singh, Kshitij Tomar

**Affiliations:** 1 Critical Care Medicine, Max Super Speciality Hospital, Vaishali, Ghaziabad, IND

**Keywords:** altered sensorium, benzodiazepines, critical care, emergency medicine, icu, immunoassay, toxicology, urine drug screening

## Abstract

Background

Urine drug screening (UDS) using immunoassay-based panels is widely implemented in acute and intensive care settings for patients presenting with altered sensorium, unexplained agitation, or suspected intoxication. The diagnostic yield of UDS, however, varies considerably across regions and time periods, and data from North India remain limited. Immunoassays are subject to limitations such as cross-reactivity and false-positive results. Consequently, longitudinal, center-specific data are essential for contextualizing test utility and optimizing clinical decision-making.

Aims and objectives

This retrospective study evaluated annual and seasonal variation in UDS positivity and characterized the spectrum of substances detected over a consecutive four-year period at a North Indian tertiary care center.

Methods

A retrospective observational study was conducted at Max Super Speciality Hospital, Vaishali, a tertiary care center in the National Capital Region (NCR), India. All unique urine drug assay panels performed between January 2022 and December 2025 were analyzed (N = 323), irrespective of patient age, sex, or clinical indication. A panel was considered positive if at least one drug analyte was detected. Data were analyzed by year and season (winter: December-February; summer: March-May; monsoon: June-September; post-monsoon: October-November). Categorical variables were compared using the Chi-squared test, with p < 0.05 considered statistically significant.

Results

Overall, 42 of 323 panels (13.0%) were positive. Annual UDS positivity was 8.33% in 2022 and 7.63% in 2023, with higher rates of 23.64% in 2024 and 26.83% in 2025. The annual distribution differed significantly across the study years (p = 0.0005). Significant seasonal variation was observed (p = 7.7 × 10⁻⁶), with the highest positivity during the monsoon (27.14%) and summer (20.83%) and the lowest during winter (3.87%). Among 52 analyte-level detections, benzodiazepines accounted for 28 of 52 detections (53.8%), followed by tricyclic antidepressants (6/52, 11.5%) and cannabinoids (5/52, 9.6%).

Conclusion

UDS positivity varied significantly across study years and showed pronounced seasonal variation, with the highest yield during the monsoon. Benzodiazepines were the most frequently detected drug class. Given the limitations of immunoassays, UDS results should be interpreted within the clinical context, and confirmatory testing should be considered when results would materially affect diagnosis or management. Future multicenter prospective studies incorporating clinical indications and definitive toxicological testing are needed to validate these findings.

## Introduction

Altered sensorium and unexplained agitation are common presentations in emergency departments and intensive care units (ICUs), with toxicological causes representing a substantial and potentially reversible proportion. Identifying the underlying etiology is challenging due to a broad differential diagnosis that includes metabolic disturbances, neurological disorders, infections, withdrawal syndromes, and toxicological causes [1,2]. Early recognition of drug intoxication can substantially affect patient management, including decisions regarding airway protection, cardiac monitoring, antidote administration, and the required level of care. Urine drug screening (UDS) is frequently employed in acute care settings due to its rapidity, relative affordability, and non-invasive nature [3]. Most UDS panels utilize immunoassay techniques and serve as screening rather than confirmatory tests, with results interpreted against assay-specific cut-off concentrations. Interpretation requires caution: false-positive results may arise from cross-reactivity with prescribed or over-the-counter medications, while false-negative results may occur due to variations in detection windows, metabolite characteristics, or assay sensitivity [4-7]. Therefore, UDS findings should always be interpreted in conjunction with the clinical context. Historically, acute poisoning in India has been predominantly linked to pesticide exposure, particularly in rural populations [8]. However, tertiary care hospitals in urban areas are increasingly managing cases related to pharmaceutical overdose, sedative-hypnotic use, and polysubstance exposure [9]. This epidemiological transition necessitates contemporary, center-specific data to guide testing practices. Additionally, seasonal variations in poisoning patterns may impact resource planning and clinical response. However, such longitudinal data from North Indian tertiary care settings remain limited. This retrospective study analyzed UDS panels conducted over a four-year period at a tertiary care center in North India. The objectives were to (1) examine annual trends in UDS positivity rates, (2) characterize the spectrum of substances detected, and (3) assess seasonal variations in UDS positivity.

## Materials and methods

Study design

This was a retrospective observational study of all laboratory records generated between January 1, 2022, and December 31, 2025. The study was approved by the Institutional Ethics Committee of Max Super Speciality Hospital, Vaishali (EC Ref. No. BHR/RS/MSSH/VSH/CRL/IEC/CCM/26-06). The requirement for individual informed consent was waived owing to the retrospective nature of the study and use of de-identified laboratory data.

Setting

The study was conducted at Max Super Speciality Hospital, Vaishali, a tertiary care hospital with more than 385 beds in the National Capital Region of India, serving a mixed urban and peri-urban population.

Study population

All UDS panels performed during the study period were reviewed. Each panel was identified by a unique laboratory barcode. When multiple analyte entries belonged to the same barcode, they were consolidated and analyzed as one panel-level record to prevent duplication. Patients could contribute more than one panel if multiple tests were performed on different dates; each panel was analyzed independently.

Inclusion criteria

All UDS panels processed by the hospital laboratory from January 1, 2022, through December 31, 2025, were eligible for inclusion, irrespective of patient age, sex, clinical indication, or requesting department. Only one panel-level record was retained for each unique laboratory barcode.

Exclusion criteria

Duplicate laboratory entries referring to the same UDS panel were excluded after consolidation based on unique laboratory barcodes. Records were also excluded when they lacked information required to determine the test date, the panel result, or both. Any UDS request performed outside the prespecified study period was ineligible for analysis.

Laboratory methodology

UDS was performed using qualitative immunoassay techniques capable of detecting the following drug classes: benzodiazepines, cannabinoids, barbiturates, amphetamines, methamphetamines, cocaine, opiates, tricyclic antidepressants (TCAs), and phencyclidine (PCP). Testing was performed according to the manufacturer's protocols. The tests were used as screening assays; routine confirmatory testing by gas chromatography-mass spectrometry (GC-MS) or liquid chromatography-tandem mass spectrometry (LC-MS/MS) was not available for positive results.

Definitions

For this study, a positive UDS panel was defined as one in which at least one analyte tested yielded a positive immunoassay result. Seasons were classified according to standard meteorological periods: winter (December-February), summer (March-May), monsoon (June-September), and post-monsoon (October-November).

Statistical analysis

Raw laboratory metadata were extracted into structured spreadsheets and analyzed using IBM SPSS Statistics, version 26.0 (IBM Corp., Armonk, NY, USA). Continuous age data were summarized using the mean and standard deviation, median and interquartile range, and range. Categorical variables were summarized as absolute frequencies with percentages. For panels with multiple positive analytes, the panel was counted once for positivity rate calculations, while each detected substance was counted separately for substance distribution analyses. The annual positivity rate was calculated by dividing the number of positive panels in a given year by the total panels tested in that year. The seasonal positivity rate was calculated similarly for each season. Associations between UDS result (positive or negative) and year, and between UDS result and season, were assessed using Pearson's chi-squared test of independence. The chi-squared statistic, degrees of freedom, and two-tailed p-value were reported. Statistical significance was defined as p < 0.05. No a priori sample size calculation was performed, as this was an analysis of all available records (complete census).

## Results

Demographic and clinical characteristics

A total of 323 unique UDS panels were included. The demographic and clinical characteristics of the study population are summarized in Table 1. The mean age was 33.1 ± 15.8 years, the median age was 28 years (interquartile range: 23-38 years), and the age range was 1-95 years. The study included 219 males (67.8%) and 104 females (32.2%). Requests originated from the outpatient department for 163 panels (50.5%), the ICU for 96 panels (29.7%), and inpatient wards for 64 panels (19.8%).

**Table 1:** Demographic and clinical characteristics of the study population (N = 323). The table summarizes participants' age, sex distribution, and the clinical location from which urine drug screening was requested. Data are presented as n (%), mean ± SD, median (IQR), or range, as appropriate. SD: standard deviation; IQR: interquartile range

Characteristic	n (%)/value
Total study population	323
Age, years	
Mean ± SD	33.1 ± 15.8
Median (IQR)	28 (23-38)
Range	1-95
Sex	
Male	219 (67.8%)
Female	104 (32.2%)
Patient category	
Outpatient department	163 (50.5%)
Intensive care unit	96 (29.7%)
Inpatient ward	64 (19.8%)

Overall and annual diagnostic yield

Overall, 42 of 323 UDS panels (13.0%) were positive for one or more substances. Annual positivity was 8.33% (8/96) in 2022, 7.63% (10/131) in 2023, 23.64% (13/55) in 2024, and 26.83% (11/41) in 2025, demonstrating a marked increase in the final two years of the study. The annual distribution of positive and negative panels differed significantly (Pearson's chi-squared test: χ² = 17.62, df = 3, p = 0.00053) (Table 2).

**Table 2:** Annual urine drug screening (UDS) diagnostic yield. Percentages in the “Total panels” column were calculated using all 323 panels as the denominator, and percentages in the “Positive panels” column were calculated using all 42 positive panels as the denominator. The positivity rate was calculated as positive panels divided by total panels within each year. Annual distributions were compared using Pearson's chi-squared test (χ² = 17.62, df = 3, p = 0.00053).

Year	Total panels, n (%)	Positive panels, n (%)	Positivity rate, n/N (%)	Test result
2022	96 (29.72%)	8 (19.05%)	8/96 (8.33%)	Pearson's chi-squared test: χ² = 17.62, df = 3, p = 0.00053
2023	131 (40.56%)	10 (23.81%)	10/131 (7.63%)	
2024	55 (17.03%)	13 (30.95%)	13/55 (23.64%)	
2025	41 (12.69%)	11 (26.19%)	11/41 (26.83%)	
Total	323 (100.00%)	42 (100.00%)	42/323 (13.00%)	

Seasonal variation in positivity

Seasonal positivity was highest during the monsoon (27.14%, 19/70) and summer (20.83%, 10/48), followed by post-monsoon (14.00%, 7/50), and lowest during winter (3.87%, 6/155). The seasonal distribution of positive and negative panels differed significantly (Pearson's chi-squared test: χ² = 26.44, df = 3, p = 7.70 × 10⁻⁶) (Table 3).

**Table 3:** Seasonal variation in urine drug screening (UDS) positivity. Percentages in the “Total panels” column were calculated using all 323 panels as the denominator, and percentages in the “Positive panels” column were calculated using all 42 positive panels as the denominator. The positivity rate was calculated as positive panels divided by total panels within each season. Seasonal distributions were compared using Pearson's chi-squared test (χ² = 26.44, df = 3, p = 7.70 × 10⁻⁶).

Season	Total panels, n (%)	Positive panels, n (%)	Positivity rate, n/N (%)	Test result
Monsoon	70 (21.67%)	19 (45.24%)	19/70 (27.14%)	Pearson's chi-squared test: χ² = 26.44, df = 3, p = 7.70 × 10⁻⁶
Summer	48 (14.86%)	10 (23.81%)	10/48 (20.83%)	
Post-monsoon	50 (15.48%)	7 (16.67%)	7/50 (14.00%)	
Winter	155 (47.99%)	6 (14.29%)	6/155 (3.87%)	
Total	323 (100.00%)	42 (100.00%)	42/323 (13.00%)	

Substance detection pattern

Among the 42 positive UDS panels, 52 analyte-level drug detections were recorded because some panels contained more than one detected drug class. Benzodiazepines were the most frequently detected drug class, accounting for 28/52 detections (53.8%), followed by TCAs at 6/52 (11.5%), cannabinoids at 5/52 (9.6%), PCP at 4/52 (7.7%), and barbiturates at 3/52 (5.8%). The remaining six detections (11.5%) were distributed among amphetamine, methamphetamine, opiates, cocaine, and 3,4-methylenedioxymethamphetamine (Table 4).

**Table 4:** Distribution of 52 analyte-level drug detections among 42 positive UDS panels. AMP: amphetamine; MET: methamphetamine; OPI: opiates; COC: cocaine; MDMA: 3,4-methylenedioxymethamphetamine; UDS: urine drug screening

Substance	Detections (n)	Percentage, n/N (%)
Benzodiazepines	28	28/52 (53.8%)
Tricyclic antidepressants	6	6/52 (11.5%)
Cannabinoids	5	5/52 (9.6%)
Phencyclidine	4	4/52 (7.7%)
Barbiturates	3	3/52 (5.8%)
Others (AMP, MET, OPI, COC, MDMA)	6	6/52 (11.5%)

## Discussion

This four-year laboratory-based study found that annual UDS positivity differed significantly across the study period, with a marked increase after 2023. Positivity also varied significantly by season and was highest during the monsoon. Benzodiazepines were the predominant detected drug class.

Annual diagnostic yield

The increase in observed positivity after 2023 is noteworthy and may reflect several possibilities. First, changes in the case mix, including a higher proportion of patients with toxicological presentations, could have contributed. Second, more selective ordering practices-where clinicians request UDS only in patients with a higher pre-test probability of intoxication-may have increased diagnostic yield. Third, the increase may reflect genuine changes in substance exposure patterns in the community served by the hospital. However, because this study used retrospective laboratory data and did not capture the clinical indication for each request, these explanations cannot be separated. An alternative explanation is that the trend might be artefactual, reflecting changes in clinician ordering behavior, laboratory threshold adjustments, or seasonal variations in testing volume. Previous work has emphasized that broad, indiscriminate immunoassay testing may have limited diagnostic value, whereas targeted testing can provide more clinically interpretable information [3]. The observed increase in positivity in the latter years of this study could also reflect clinicians becoming more judicious in their test ordering, although this remains speculative.

Substance patterns and clinical implications

Benzodiazepines were the most frequently detected drug class, accounting for 53.8% of all detections. This finding is clinically plausible given their widespread therapeutic availability, common non-medical use, and frequent involvement in mixed-drug exposure [9,10]. The high prevalence highlights the importance of clinicians being vigilant for benzodiazepine intoxication, particularly in patients presenting with sedation, respiratory depression, or withdrawal. TCAs, detected in 11.5% of substance detections, are clinically important because overdose can cause sodium-channel blockade, leading to cardiac arrhythmias, hypotension, seizures, and central nervous system depression [11]. Recognition of TCA exposure should prompt immediate electrocardiographic monitoring and consideration of sodium bicarbonate therapy. Cannabinoids were detected in 9.6% of cases. While cannabis intoxication typically causes mild symptoms, it can precipitate anxiety, paranoia, and psychosis in susceptible individuals, and may also contribute to polysubstance toxicity [10]. PCP was identified in 7.7% of detections. True PCP exposure can cause severe agitation, altered mental status, hyperthermia, trauma, and rhabdomyolysis [12]. However, given the lack of confirmatory testing, cross-reactivity with other substances must be considered [4]. The detection of PCP in a North Indian setting is noteworthy and warrants further investigation. Barbiturates, detected in 5.8% of cases, are encountered less frequently in clinical practice today but remain available in some settings, particularly in institutional or chronic pain populations. Their detection should prompt assessment for respiratory depression and withdrawal risk. The detection of multiple substances in some panels (as suggested by 52 detections from 42 positive panels) underscores the common occurrence of polysubstance exposure, which may complicate clinical management and require a multiagent approach to treatment.

Seasonal findings

The observed clustering of positive UDS panels during the monsoon (27.14%) and summer (20.83%), with notably lower yield during winter (3.87%), indicates significant seasonal heterogeneity in this center's testing yield. Prior poisoning studies have also reported seasonal variation, although the direction and magnitude differ across settings and poison types [13]. In the Indian context, the monsoon season has been associated with increased agricultural activity and pesticide exposure in rural populations, although this may be less relevant in an urban tertiary care setting. Alternative explanations include the following: Exposure opportunities: increased social gatherings, travel, and festivals during summer and monsoon may lead to higher rates of substance use. Psychosocial stressors: seasonal changes in mental health symptoms, including anxiety and depression, may influence substance use patterns [13]. Healthcare-seeking behavior: patients may be more likely to present to emergency services during extreme weather conditions, potentially altering the case mix. Clinician test-ordering practices: seasonal variations in the threshold for ordering UDS cannot be excluded. These mechanisms were not measured in the present study, so the seasonal association should be interpreted as exploratory rather than causal. Nevertheless, the finding has potential implications for resource planning, suggesting that higher staffing levels, increased availability of antidotes, and greater readiness for toxicology consultations may be warranted during the monsoon and summer months.

Interpretation of immunoassay findings

Routine UDS immunoassays detect drug classes using assay-specific antibodies and cut-off concentrations. A positive result indicates presumptive detection and does not by itself establish intoxication, dose, timing, or causality. False-positive and false-negative findings are well recognized. For example, the benzodiazepine immunoassay may cross-react with certain non-benzodiazepine compounds, while the amphetamine assay may react with over-the-counter decongestants such as pseudoephedrine [4,5]. Similarly, the PCP immunoassay, while less commonly used, is known to cross-react with dextromethorphan and other agents [4]. In the absence of confirmatory testing, clinicians should interpret UDS results cautiously, using them as one component of a comprehensive clinical assessment that includes history, physical examination, and other laboratory findings. Definitive testing using chromatographic mass spectrometric methods (GC-MS or LC-MS/MS) is preferable when the result has important diagnostic, legal, or therapeutic consequences, including potential medicolegal implications or decisions regarding antidote administration and level of care [1,3-7,14].

Limitations

This study has several limitations. Its retrospective, single-center design limits generalizability. The analysis was based on laboratory panels rather than complete clinical records, so indications for testing, prescribed medications, toxidromes, treatment changes, and patient outcomes could not be assessed. Immunoassay results were not routinely confirmed by GC-MS or LC-MS/MS, creating the possibility of false-positive and false-negative classification. Clinician-directed ordering and changes in ordering behavior may have influenced annual and seasonal yields. The subgroup counts were modest, and the seasonal analysis was exploratory. Finally, analyte-level detections do not necessarily represent distinct exposures because a single positive panel can contain multiple detected drug classes.

## Conclusions

UDS positivity differed significantly across years and seasons at this North Indian tertiary care center, with higher annual yield after 2023 and the highest seasonal yield during the monsoon. Benzodiazepines were the most frequently detected drug class. These findings describe local testing patterns but should not be interpreted as proof of population-level prevalence or seasonally caused exposure. UDS should remain an adjunct to clinical assessment, and confirmatory testing should be considered when a presumptive result could materially affect diagnosis or management. Multicenter prospective studies incorporating clinical indications, outcomes, and definitive toxicological testing are needed.
